# Perspectives for the Use of Fucoidans in Clinical Oncology

**DOI:** 10.3390/ijms231911821

**Published:** 2022-10-05

**Authors:** Mikhail V. Kiselevskiy, Natalia Yu. Anisimova, Nadezhda E. Ustyuzhanina, Dmitry Z. Vinnitskiy, Alexandra I. Tokatly, Vera V. Reshetnikova, Irina O. Chikileva, Irina Zh. Shubina, Kirill I. Kirgizov, Nikolay E. Nifantiev

**Affiliations:** 1 N.N. Blokhin National Medical Research Center of Oncology, Ministry of Health of Russia, 24 Kashirskoe Sh., Moscow 115478, Russia; 2 Center for Biomedical Engineering, National University of Science and Technology MISIS, Leninsky Prospect 4, Moscow 119049, Russia; 3 N. D. Zelinsky Institute of Organic Chemistry, Russian Academy of Sciences, Leninsky Av., 47, Moscow 119991, Russia

**Keywords:** fucoidan, antitumor activity, supportive therapy, P-selectin

## Abstract

Fucoidans are natural sulfated polysaccharides that have a wide range of biological functions and are regarded as promising antitumor agents. The activity of various fucoidans and their derivatives has been demonstrated in vitro on tumor cells of different histogenesis and in experiments on mice with grafted tumors. However, these experimental models showed low levels of antitumor activity and clinical trials did not prove that this class of compounds could serve as antitumor drugs. Nevertheless, the anti-inflammatory, antiangiogenic, immunostimulating, and anticoagulant properties of fucoidans, as well as their ability to stimulate hematopoiesis during cytostatic-based antitumor therapy, suggest that effective fucoidan-based drugs could be designed for the supportive care and symptomatic therapy of cancer patients. The use of fucoidans in cancer patients after chemotherapy and radiation therapy might promote the rapid improvement of hematopoiesis, while their anti-inflammatory, immunomodulatory, and anticoagulant effects have the potential to improve the quality of life of patients with advanced cancer.

## 1. Introduction

**Figure 1:**
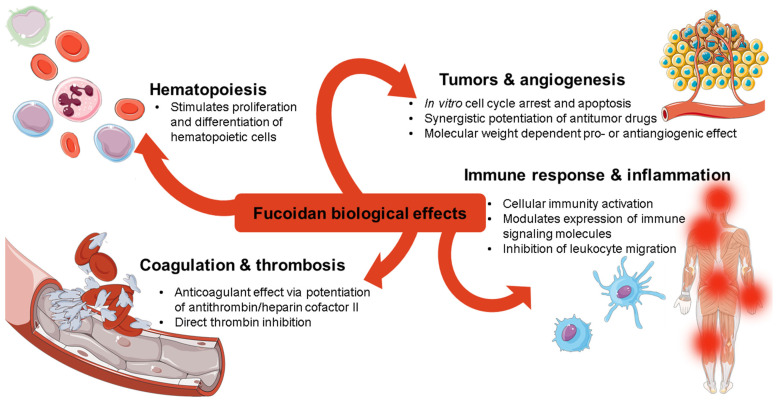
Main types of biological activities of fucoidans.

Fucoidans are natural sulfated polysaccharides present in the cell wall matrices of brown algae and a number of marine invertebrate tissues [1,2,3], which possess a wide range of biological activities (Figure 1) and have been considered as promising antitumor agents [1,4,5]. These biopolymers typically consist of linear backbone chains formed by α-L-fucopyranose units linked via (1→3)- or alternating (1→3)/(1→4)-bonds occasionally branched with L-fucopyranose, D-glucuronic acid, D-galactopyranose, and other mono- and oligosaccharide substituents, all of which can be O-sulfated in various positions [1,3]. Since their discovery in 1913 [6], there has been extensive research into their various physiological effects. Over the past decade, the number of publications and clinical trials in this area has increased significantly, indicating high interest in the biological functions and clinical use prospects of these biopolymers [1,4,5].

Fucoidans are currently being investigated as potential drugs for the supportive care of cancer patients, as well as for their use to complement conventional antitumor treatments [7,8]. Despite the significant amounts of research, only a few fucoidan-based pharmaceutical products have been designed to this day and, at present, fucoidan derivatives are mainly used as food additives [9]. This review presents an analysis of the literature on the antitumor properties of natural and modified fucoidans and the prospects of their clinical use as supportive care with antitumor therapy.

## 2. Antitumor Activity

The antitumor activity of various fucoidans and their derivatives has been demonstrated on tumor cells of different histogenesis, including lung, breast, liver, colon, prostate, and bladder cancer cells [10,11,12]. Research has shown that fucoidans altered the cell cycle resulting in increased numbers of cells in sub-G_0_ or G_1_ phase [13]. In particular, low- molecular-weight fucoidans arrested the cell cycle of human colon cancer cells in phase G_1_ in a process associated with p53-independent apoptosis [14]. Fucoidans have also been found to induce autophagy in human gastric cancer cells [15]. Fucoidans could induce apoptosis of tumor cells via TLR4-dependent activation of caspase-3 and therefore enhance the effectiveness of antitumor drugs [16]. Studies on the hepatocellular carcinoma cell model showed that fucoidans inhibited the invasion of tumor cells. The mechanism of this effect is associated with NDRG-1/Cap43-dependent regulation of the inhibitor of the DNA-binding protein ID1 that regulates the cell cycle and differentiation [17]. In particular, ID1 is associated with oncogenesis, cellular aging, as well as cell proliferation and survival. Overexpression of ID1 was registered in many types of cancer as having a stimulating effect on tumor progression [18].

The antitumor action of fucoidans is most often connected with the activation of caspase-dependent apoptosis pathways. Research has shown that fucoidans induced apoptosis and reduced telomerase activity by inhibiting the activation of the PI3K/Akt signaling pathway. A number of experimental studies confirming the antitumor activity of fucoidans in vitro and in vivo are summarized below in Table 1. A significant part of the research evaluated the effectiveness of fucoidans in combination with chemotherapy drugs. In particular, it was shown that fucoidan from the algae *Cladosiphon novae-caledoniae* had a synergistic effect with such antitumor drugs as tamoxifen, cisplatin, and paclitaxel, intensifying the cell cycle arrest, apoptosis, and inhibition of proliferation in MCF-7 breast cancer cells. The combined use of fucoidan with antitumor drugs 5-fluorouracil, cetuximab, and avastin had a synergistic inhibitory effect on metastases of colorectal cancer in experimental animals [19].

Various studies on mice with tumor grafts have shown the antitumor and antimetastatic effects of fucoidans. Thus, it was demonstrated that the repeated administration of an extract from *Fucus evanescens* (three injections at a dose of 10 mg/kg) led to regression of metastases by 29%, but did not increase the inhibition of tumor growth caused by cyclophosphamide [20]. Fucoidan from *Laminaria japonica* inhibited tumor growth by 33.7–47% [21]. One of the latest studies on a human lung cancer xenograft model in immunodeficient mice demonstrated a similar antitumor activity of sulfated galactofucans from *Sargassum thunbergii* and their derivatives. The inhibiting effect on tumor growth was observed to be 31–45% [22].

Only a few studies registered an antitumor effect of fucoidans above 50% at doses of 10–20 mg/kg [23,24]. Among these was the fucoidan from *Ecklonia cava* (ECF), administered intranasally in mice, which promoted the activation of dendritic cells, natural killer cells (NK) and T cells. In addition, it was found that intranasal injection of ECF enhanced the antitumor effect of PD-L1 antibodies on melanoma B16 and carcinoma CT-26 in lung metastases, showing that ECF could function as an adjuvant enhancing the immunotherapeutic effect of immune checkpoint inhibitors against metastatic lung cancer [25].

A recent meta-analysis of 23 publications suggested that fucoidans could inhibit tumor growth and metastatic process in various animal models. However, the authors noted the low quality study design of the included reports potentially leading to systematic error and heterogeneity, which might affect the accuracy of the results [26]. Since most studies showed the fucoidan-associated inhibition of tumor growth to be less than 50% (ED50), these effects, in accordance with the criteria of antitumor effect estimation, seem doubtful. Therefore, no final conclusion as to the antitumor activity of sulfated poly- and oligosaccharides has yet been drawn [27].

A prospective double-blind experimental study on 100 dogs with cancer evaluated the effect of low-molecular weight fucoidan from brown algae *L. japonica* on the quality of life of animals undergoing antitumor chemotherapy (68 subjects received fucoidan, 32—placebo). Treatment with this polysaccharide preparation was found to be safe and improved some quality of life parameters, but had no effect on the results of laboratory and clinical tests [28].

In addition to numerous studies of fucoidan antitumor activity in animals with spontaneous and engrafted tumors, several clinical trials were completed. The first prospective randomized double-blind controlled clinical trial evaluating the efficacy of low-molecular-weight fucoidan from *Sargassum hemiphyllum* as adjuvant therapy included 58 patients with metastatic colorectal cancer undergoing FOLFIRI therapy with the 28 patients of the main group receiving oral powder of fucoidan. The summarized rate of complete and partial response and stabilization of the disease was significantly higher in the main group compared to the control group (92.8% vs. 69.2%; *p* = 0.026). However, overall survival, severity of adverse events and quality of life had no statistical differences between the main and control groups [29].

A study involving 20 patients with advanced cancer, who received a fucoidan preparation (product by Daiichi Sangyo Co., Tokyo, Japan, parent algae is not indicated) at a dose of 400 mL/day per os for 4 weeks, revealed a decrease in the mononuclear leukocyte production of pro-inflammatory cytokines after 2 weeks of therapy. However, the effect was short-term and the secretion of inflammatory mediators had an obvious tendency towards restoration by the end of the 4 weeks. Other immunological parameters, as well as the patients’ quality of life did not change during the treatment [30].

Several randomized studies evaluating the effect of fucoidan on the quality of life in patients receiving chemotherapy have shown inconclusive results, which may be explained by the differences in the patients’ status or the types of the fucoidan-based drugs used [31]. Similarly, some positive dynamics in the condition of cancer patients were reported in response to the use of low-molecular-weight fucoidan extracts [32]. The effectiveness of fucoidan adjuvant therapy in cancer patients has been analyzed in the course of 4 clinical trials that included 118 patients with metastatic colorectal and gastric cancers [33]. Two studies showed increased overall survival in patients receiving fucoidan. Positive, but insignificant effects of fucoidan on the disease control rate, inflammatory markers, nutrition status, and fatigue were also observed. However, according to the authors, the published data do not provide sufficient grounds to consider fucoidans effective antitumor agents or modifiers of chemotherapeutic drugs. Nevertheless, the wide range of biological activities exhibited by this class of compounds makes them a potential base for the development of drugs for the supportive care and palliative therapy of cancer patients.

## 3. Use in Adoptive Cell-Based Antitumor Immunotherapy

Adoptive transfer of antigen-specific T cells is a promising approach to cancer treatment [34,35]. Studies have shown that genetic modification of T cells by introducing chimeric antigen receptors (CAR) increases their effectiveness [36]. CAR-T cells targeting CD19 have shown excellent clinical results against B-cell malignancies [37]. However the effectiveness of T cell constructs remains low in the case of solid tumor treatment [38]. One of the problems of adoptive CAR-T cell immunotherapy is the induction of a suppressor subpopulation of T-regulatory cells during ex vivo expansion of lymphocytes in the presence of IL-2. To address CAR-T cell therapy limitations, IL-2 delivery microcapsules consisting of fucoidan, a specific IL-2 binding glycosaminoglycan, and poly-L-lysine (FPC^2^) have been developed. FPC^2^ loaded with IL-2 showed a higher biological activity in ex vivo expansion from cytotoxic T cells than from Treg lymphocytes. A single intra-tumor administration of the FPC2/IL-2 complex with injectable gel had a favorable effect on the subpopulation ratio of tumor-infiltrating leukocytes as a result of the enhanced expansion of cytotoxic T lymphocytes and decreased number of myeloid subpopulations. This approach represents a new method in TCR-engineered T-cell therapies targeting solid tumors [39].

Fucoidan is known to have an immunomodulatory effect [40]. It was shown that the fucoidan from *Fucus vesiculosus* mediated in vivo activation of dendritic cells (DCs) in the spleen and lymph nodes of mice, thus triggering antitumor immune reactions, and contributed to the maturation of human and murine DCs. Systemic administration of this polysaccharide induced increased expression of CD40, CD80, and CD86 on the DCs of the spleen and enhanced production of IL-6, IL-12, and TNF-α. Fucoidan also promoted the proliferation of IFN-γ-producing Th1 cells [41,42]. Another study demonstrated that the fucoidan from *E. cava* (ECF) caused the activation of bone marrow DCs in vitro and splenic DCs in vivo. Combined treatment with ECF and autoantigen resulted in inhibition of CT-26 carcinoma growth in mice due to the induction of antigen-specific immunity [43], which suggests that fucoidan can be used as an adjuvant in antitumor DC vaccines.

## 4. Antiangiogenic Effect

Angiogenesis involves the proliferation, differentiation, and migration of mature endothelial cells, and is regulated by various endothelial angiogenic factors, including platelet growth factor, vascular endothelial growth factor (VEGF) and fibroblast growth factor (FGF) [44]. Since tumor angiogenesis supports the growth, progression, and metastasis of the tumor, researchers have a special interest in developing antiangiogenic strategies to inhibit tumor vascularization. Traditional treatments use antiangiogenic drugs to block the activity of proangiogenic factors, which frequently exhibit debatable efficacy, while the long-term therapy with these drugs leads to the development of tumor resistance [45].

Given these limitations, the search for alternative ways of inhibiting angiogenesis is still an important issue. Accordingly, many types of fucoidans were tested as inhibitors of angiogenesis due to their known ability to regulate the expression of VEGF and inhibitor-1, an activator of endothelial cell plasminogen. In addition, fucoidan suppresses the migration of endothelial cells by interacting with matrix metalloproteinases and chemokine CXCL12 [46,47,48].

Currently, there are contradictory data on how fucoidan structure affects antiangiogenic activity. In general, higher degrees of sulfation tend to favor antiangiogenic activity [46]. In vitro experiments showed oversulfated fucoidan to inhibit angiogenesis in endothelial cells in 3D cultures [49]. Fucoidan isolated from brown algae *Sargassum fusiforme* inhibited the development of microvessels by human endothelial cells; however, the effect was not dose-dependent [50]. On the other hand, low-molecular-weight fucoidans may have proangiogenic activity due to their ability to modulate heparin-binding growth factors, such as FGF-2 [51]. Indeed, it has been shown that the effect of fucoidans on angiogenesis largely depends on their molecular weight: antiangiogenic activity is associated with high-molecular fucoidans, whereas low-molecular fractions can function as proangiogenic agents [52].

## 5. Anticoagulant and Antithrombotic Activities

Thrombosis is a frequent complication of cancer and is considered to be one of the main causes of death of cancer patients [53]. Oncological disease is known to increase the risk of deep vein thrombosis and pulmonary embolism by 4–7 fold. One treatment option for hypercoagulation and venous thromboembolism is heparin (low-molecular-weight heparins)–one of the most widely used intravenous anticoagulants, exhibiting a unique polyanionic structure [54,55]. However, extensive heparin treatment is limited due to its hemorrhagic effect and the risk of heparin-induced thrombocytopenia [56]. Evidently, it is necessary to expand the number of anticoagulants by developing effective drugs with fewer adverse effects. Fucoidans exhibit anticoagulant and antithrombotic activity mediated by heparin cofactor II and other blood-clotting factors [57]. These effects are also associated with ability of sulfated polysaccharides to potentiate the interaction of thrombin with antithrombin (ATIII) or heparin cofactor II (HCII) Other pathways include direct inhibition of thrombin and factor Xa [58]. Fucoidans with a higher molecular weight tend to show a more pronounced anticoagulant effect while a certain sulfation rate is essential for anticoagulant activity [59,60].

A single blind clinical study evaluated the anticoagulant activity of fucoidan from *Undaria pinnatifida*, with 10 subjects receiving 3 g of fucoidan capsules for 12 days (the 10 subjects in the control group received guar gum capsules). Despite the fact that preliminary in vitro studies had revealed a pronounced anticoagulant activity of the studied fucoidan, no effect on hemostasis in vivo was apparent, probably due to low intestinal absorption [61].

## 6. Immunoregulatory Activity

Many pharmacological effects of fucoidan including antiviral and antitumor activity are largely explained by its ability to modulate cellular immunity [62,63,64]. It is assumed that fucoidans bind to various receptors, such as the Toll-like receptors on dendritic cells and macrophages and monocytes, inducing the release of cytokines and chemokines necessary for an immune response [40].

Numerous studies have confirmed the effect of fucoidans on immune regulation. In particular, fucoidan from *F. vesiculosus* enhanced the production of TNF-α and IL-6 by peritoneal macrophages [65]. In addition, fucoidan has been found to contribute to the improvement of the Th1/Th2 immune balance [43].

A randomized double-blind parallel placebo-controlled cohort study evaluated the effect of fucoidan from *Cladosiphon okamuranus* on the activity of human NK cells. The main cohort included 20 subjects who received fucoidan orally in a single dose of 3 g for 12 weeks. NK cell activity was shown to be significantly higher in the cohort receiving fucoidan than in the reference cohort [66]. Fucoidan also activated the growth of T and B cells in the spleen. It was found [67] that fucoidans with different molecular weights had varying effects on the proliferation of NK and T cells, with high-molecular-weight fucoidan increasing the ratio of cytotoxic T-cells [65]. In addition, fucoidans were able to activate phagocytes, including macrophages [68].

## 7. Anti-Inflammatory Activity

Chronic inflammation can stimulate the development and progression of tumors [69]. The inflammatory reaction in cancer patients may result from intensive antitumor treatment, such as extended operations and chemotherapy, and it is mediated by increased levels of inflammatory mediators and reactive radicals, particularly NO [70]. An important role in the development of the inflammatory reaction in cancer patients is the disruption of the intestinal permeability, resulting in the translocation of bacterial toxins into the systemic circulation [71].

Experimental studies have shown that fucoidans have anti-inflammatory activity, reducing the levels of inflammatory mediator and NO release induced by bacterial lipopolysaccharides. Fucoidans exhibit anti-inflammatory activity via their ability to inhibit the migration of leukocytes into the tissues of the inflammatory site [47,72]. Oral fucoidan application reduced the elevated levels of pro-inflammatory cytokines, including TNF-α, IL-1β, and IL-6 in a murine colitis model [73].

Fucoidans have been found to restore intestinal barrier function by inhibiting TLR4/NF-kB signaling pathway [74]. It has also been reported that fucoidans could restore the tissues of ulcerative and inflammatory precancerous lesions of the gastrointestinal tract due to their ability to regulate the immune response and reduce inflammation [75]. Fucoidans can reduce inflammatory reaction symptoms in cancer patients [30]. It has also been demonstrated that fucoidans could synergistically enhance the effectiveness of anti-inflammatory drugs [66].

In recent years, studies have shown fucoidans to exhibit pharmacological activity in inflammatory bowel disease (IBD), which is associated with the destruction of the intestinal epithelial cells and the subsequent increase of paracellular permeability. In particular, the application of fucoidan can diminish mucosal damage and crypt destruction in the murine chronic colitis model [76], while the fucoidan from *C. okamuranus* suppressed the expression of pro-inflammatory cytokine IL-6 in the epithelial cells which improved chronic colitis in mice with IBD [77].

A randomized study on the anti-inflammatory effect of wheat peptides and fucoidan (WPF) was performed including 106 patients: 53 in the control group and 53 in the study group receiving WPF orally once a day for 45 days. The analysis of the results showed that the use of WPF reduced the damage of the gastric mucosa in 70% of subjects and significantly reduced the severity of dyspeptic events [78,79,80].

## 8. Stimulation of Hematopoiesis

Chemo- and radiation antitumor therapies cause a number of serious adverse effects, such as disorders of hematopoiesis, inhibition of bone marrow activity and immunosuppression, which require pharmacological correction [81,82]. Currently, colony-stimulating factors G-CSF and GM-CSF are used for these purposes [83]. However, these agents have a stimulating effect only on leukocytes and are insufficient in the case of pancytopenia. In addition, the colony-stimulating factors used for long-term treatment can trigger proliferation of tumor stem cells by stimulating pro-carcinogenic immune cells such as M2 macrophages, myeloid suppressor cells and regulatory T cells [84]. Thus, the search for new drugs to stimulate hematopoiesis in cancer patients is still of high interest.

Fucoidans and other natural sulfated polysaccharides are potential candidates for this role. Similar to preparations of microbial origin, they can induce emergency hematopoiesis [85], however, unlike bacterial derivatives, fucoidans do not display pathogenicity or general toxicity. A series of experimental studies demonstrated that the use of fucoidans and their derivatives led to the rapid recovery of all hematopoiesis processes in the model of cyclophosphamide-induced hematopoiesis inhibition in mice [86]. These studies indicate that the mechanisms of the hemostimulating effect of fucoidans are based on stimulating the proliferation and differentiation of hematopoietic cells [87].

## 9. Stimulation of Intestinal Microbiota

Recent studies have demonstrated that the microbiota of the gastrointestinal tract has a significant impact on immune homeostasis and plays an important role in the transplantation of allogeneic hematopoietic stem cells (allo-HSCT) [88]. Metabolites of the intestinal microflora can stimulate regulatory T-lymphocytes (Treg) supporting immune tolerance [89]. Patients receiving immunosuppressive therapy and antibiotics in the post-transplant period often have deviations in the homeostasis of the intestinal microbiota [90,91].

Decreased numbers of the bacteria *Lachnospiraceae* and *Blautia* and increased numbers of *Enterobacteriaceae* correlate with the development of the acute graft-versus-host disease (aGVHD) [92]. It has been suggested that gut microbiota can influence the aGVHD development by maintaining the Treg/Th17 balance [93]. Studies found that low diversity of microbiota was an independent risk factor for aGVHD [94]. Therefore, normalizing the composition of the intestinal microflora in patients after allo-HSCT is an important issue for the prevention of aGVHD. Natural fucoidan and its derivatives were recommended as a potential probiotic for this purpose. Studies have demonstrated favorable modulation of the intestinal microbiota by fucoidans: dietary fucoidans were found to promote the formation of a balanced intestinal microbiota, and significantly reduce the antigenic load and inflammatory process by lowering the level of lipopolysaccharide-binding protein in the serum [95]. These results suggest fucoidans’ potential as a modulator of the intestinal microbiota in patients after allo-HSCT.

## 10. Fucoidan-Based Nanoparticles for Antitumor Therapy and Cancer Diagnosis

The clinical studies described above evaluated the therapeutic potential of fucoidans for the treatment of cancer patients as administered in an oral dosage form. However, the researchers consistently observed low bioavailability and high clearance of fucoidans associated with this way of administration [96], which implies serious limitations for their use as antitumor drugs [97]. A promising approach to overcoming this limitation is introducing fucoidan-based nanoparticles with specified characteristics that can be changed to adjust the parameters of pharmacokinetics, as well as accumulation of fucoidan derivatives in the tumor due to binding with P-selectin [98].

Nanoparticles obtained from *F. vesiculosus* fucoidan (FuNP) with an average size of about 210 nm have been designed recently [99]. In vitro experiments showed rapid internalization of FuNPs in breast cancer cells (MDA-MB-231 cell line) and the cytotoxic activity of the nanoparticles was reported to be 7 times higher compared to the original fucoidan. Studies on mice with inoculated breast cancer tumors of the MDA-MB-231 line showed that FuNPs caused ~2.5 fold stronger inhibition of tumor growth compared to that of free fucoidan. The researchers also observed an antimetastatic effect with the use of nanoparticles. The nanoparticles were found to be well tolerated and did not cause adverse effects or morphological changes in the internal organs of the treated animals.

Fucoidan-based nanoparticles can also be viewed as a perspective platform for creating site-directed delivery systems of hydrophobic compounds. For this purpose, nanoparticles were constructed from chitosan and fucoidan, and were used as nanocarriers for the targeted delivery of antitumor drugs [100]. Nanoparticles of this type increase the solubility of chemotherapy drugs, their blood circulation, and accumulation in tumors [101]. A drug delivery system based on fucoidan-chitosan nanobeads was also used to reduce the toxicity of antitumor drugs and improve their effectiveness [102]. An evaluation of the activity of nanoparticles loaded with gemcitabine (Gem) on human breast cancer cells (MDA-MB-231) showed that the cytotoxicity was almost 25% higher compared with free Gem [103].

Another model for the delivery of hydrophobic substances to the tumor using chitosan-fucoidan nanoparticles has been studied with Piperlongumin (PL), which is a new type of pro-oxidant drug that induces cancer-specific apoptosis by enhancing intracellular reactive oxygen species (ROS). This drug displays poor solubility [104] and practically has no clinical use, but nanoparticles based on chitosan and fucoidan (CS-FNP) effectively included PL and increased its solubility in water and therefore its bioavailability. PL-loaded CS-FNP nanoparticles (PL-CS-FNP) showed higher cytotoxicity against PC-3 prostate cancer cells compared with free PL. The authors also reported selective anticancer activity of PL-CS-FNP nanoparticles demonstrating higher cytotoxicity against PC-3 tumor cells compared with that of non-transformed hDFB cells [105]. It should however be noted that the use of chitosan-fucoidan nanoparticles for the delivery of antitumor drugs may have limitations at pH above 6.5 due to chitosan deprotonation leading to decreased solubility in water [106].

Nanobeads based on fucoidan-protein complexes were investigated for their use as drug-carrying systems. Self-assembling nanoparticles of fucoidan from *L. japonica* and a cationic polypeptide protamine were studied as doxorubicin carriers and found to have an improved inhibitory effect on metastatic breast cancer cells (MDA-MB-231 line) potentially associated with P-selectin recognition of fucoidan chains which promotes endocytosis [107]. A study of nanoparticles with resveratrol based on the same type of fucoidan and zein (a natural protein of plant origin) showed a similar effect [108].

Recent studies have expanded the use of fucoidan and chitosan to improve the effects of metal nanoparticles (gold, silver or iron oxide) for the diagnosis and therapy of cancer [109,110]. To ensure the stability of metal nanoparticles in a biological environment, they are coated with hydrophilic polymers. Silver nanoparticles coated with chitosan and fucoidan complex showed effective anticancer activity in human cervical cancer cells HeLa [111]. Gold nanoparticles coated with fucoidan from *F. vesiculosus* and loaded with doxorubicin (DOX-Fu AuNP) inhibited the proliferation of human breast cancer cells at a concentration of 3–35 μg/mL at 24 h. DOX-Fu AuNP were able to induce both early and late apoptosis depending on concentration. DOX -Fu AuNP nanoparticles were also used as a contrasting agent for in vitro photoacoustic imaging of breast cancer. This indicates that nanoparticles of this type have the potential to be used widely in both drug delivery and diagnosis [112].

A large number of human tumors express P-selectin which might be considered a diagnostic marker and a target for drug delivery to tumor sites, including metastases [113]. Drug delivery systems were constructed of nanocarriers and fucoidan-based biovectors with P-selectin affinity to target tumor cells. These nanosystems containing several anticancer drugs such as paclitaxel and doxorubicin were evaluated as to their antitumor efficacy and adverse effects. Studies on the distribution of fucoidan-based nanoparticles from *F. vesiculosus* in mice with tumors expressing P-selectin revealed selective accumulation of the nanodrug in the tumor microenvironment within 24 h after injection. Targeting P-selectin increased the inhibition of tumor growth in mice by 2.8 fold compared to nanoparticles that did not contain a targeting vector [114].

Low-molecular-weight fucoidan from *Ascophyllum nodosum* [115] was used in the design of a series of P-selectin-directed contrasting agents for the visualization of inflammation sites in the human vasculature [116,117,118]. Additionally a ^68^Ga complex of fucoidan from *F. vesiculosus* was used for tracing advanced atherosclerotic plaques with high expression of P-selectin in the connected endothelium via PET imaging, successfully discriminating them from endothelium overlying inactive fibrous plaques [119]. Apart from evaluating atherosclerotic plaques, this approach to PET imaging might also be investigated for tumor diagnostics, given that a large number of human tumors, unlike normal tissues, exhibit P-selectin overexpression. Taking advantage of P-selectin targeting the fucoidan from *U. pinnatifida* has already been used to design a theranostic nanogel containing chlorin e6 photosensitizing residues, which is a unique agent with the dual functions of tumor imaging and antitumor photodynamic therapy [120].

Finally, *F. vesiculosus*-derived fucoidan vectors attached to the surface of dextran coated superparamagnetic iron oxide nanoparticles have recently been reported on as MRI contrasting agents and for magnetic hyperthermia therapy [98]. The results showed that modifying particles with fucoidan prolongs their circulation time and prevents rapid clearance via the reticuloendothelial system. This observation should incentivize the extensive investigation and implementation of fucoidans as diagnostic contrasting agents and drug delivery systems for clinical oncology.

## 11. Perspectives

Despite numerous reports on the biological investigations of fucoidans and studies into their antitumor activity in experiments on tumor cells and transplants (xenografts) in mice, clinical studies have shown that these compounds are unlikely to be effectively used as antitumor drugs (selected results are collected in Table 1). Experimental studies and clinical trials do not suggest an unambiguous conclusion as to the antitumor activity of fucoidan compounds, though studies have repeatedly demonstrated pleiotropic effects of fucoidans. Accordingly, researchers are currently discussing the potential of fucoidans as additional agents for antitumor treatment, as well as their use for the supportive care and palliative therapy of cancer patients. It has been suggested that fucoidans may play a role in reducing adverse effects and enhancing the clinical efficacy of conventional therapeutic methods. A summary of the reported clinical trials (including veterinary trials) of fucoidans towards the development of anticancer drugs is presented in Table 2.

Another fundamental problem limiting the therapeutic applicability of fucoidans is their irregular structure due to varying content of sulfate groups and other substituents, as well as variable molecular weights within fucoidan preparations. This is incompatible with the requirements of GMP-graded manufacturing and causes the problems even at the stage of pre-clinical investigations, including the study of pharmacokinetics and pharmacodynamics [121].

There are also difficulties in comparing the data obtained by different authors due to varying algal raw materials, as well as methods of extraction and purification of fucoidans. It should be also noted that possible impurities can make an important contribution to the biological activity of fucoidans. In particular, products of commensal Gram-negative bacteria in brown algae, are often disregarded [122,123,124], even though it is well known that pathogen-associated products (particularly bacterial endotoxin) activate Toll-like receptors and possess significant immunostimulating activity producing a dual effect on tumor growth: on the one hand, they can activate the antitumor immune response and inhibit tumor progression, while on the other hand, inducing inflammatory reactions of the tumor microenvironment and stimulating the proliferation of malignantly transformed cells, as well as fostering tumor evasion from the immune response [125,126,127,128].

**Table 1:** Antitumor activity of fucoidans.

Type of Cancer	Study	Source of Fucoidan	Mechanism of Action	Refs.
Acute leukemia	In vitro	*F. vesiculosus*	Induction of apoptosis	[129]
Lymphoma	In vitro and in vivo	*F. vesiculosus*	Oral administration of fucoidan inhibited tumor growth	[130]
Head and neck cancer	In vitro and in vivo	*F. vesiculosus*	Injection of fucoidan-based nanoparticles inhibited tumor growth	[131]
Nasopharyngeal carcinoma	In vitro and in vivo	*L. japonica*	Fucoidan injection inhibited tumor growth and induced apoptosis	[21]
Oral cancer	In vitro	*F. vesiculosus*	Caspase-dependent apoptosis	[132]
Bladder cancer	In vitro	*F. vesiculosus*	Arrest of the cell cycle in the G_0_/G_1_ phase. Induction of apoptosis	[133]
Melanoma	In vitro	*Sargassum henslowianum* *C. agardh* and *F. vesiculosus*	Induction of apoptosis, activation of caspase-3	[134]
Hepatocellular carcinoma	In vitro	*C. okamuranus*	Arrest of the cell cycle in the G_0_/G_1_ phase	[135]
Breast cancer	In vitro	*C. novae-caledoniae*	Combination of fucoidan with tamoxifen, cisplatin or paclitaxel inhibits cell growth, mediates cellular apoptosis and cell cycle arrest in human breast cancer cells MCF-7/MDA-MB-231	[136]
Pancreatic cancer	In vitro	*Turbinaria conoides*	Inhibition of cell proliferation and induction of apoptosis of pancreatic cancer cells	[137]
Lung cancer	In vitro	*F. vesiculosus*	Inhibition of tumor cell migration and invasion	[138]
	In vitro	*T. conoides*	Induction of apoptosis	[138]
Hepatocellular carcinoma	In vitro	*Padina pavonica and Jania rubens*	Fucoidan extracts decreases the number and viability of Hep-G2 cells	[139]
Ehrlich ascites carcinoma (EAC)	In vivo	*P. pavonica and* *J. rubens*	Fucoidan extracts reduced the number and viability of EAC tumor cells	[139]
Prostate cancer	In vitro	*F. vesiculosus*	Attenuation of the motility of docetaxel-resistant DU/DX50 cells by binding to P-selectin, downregulation of IL-1R, inactivation of NF-κB and reduction in Cox2 expression	[140]

**Table 2:** Clinical trials with the use of fucoidans.

Patients	Study	Source of Fucoidan	Clinical Effects	Refs.
100 dogs with a cancer treated with chemotherapy.	Double-blind case control study.	*L. japonica*.	Treatment with fucoidan was safe and improved some of the quality of life metrics.	[28]
54 patients with metastatic colorectal cancer.	Prospective, randomized, double-blind, controlled trial.	Low-molecular-weight fucoidan derived from *S. hemiphyllum.*	Fucoidan combined with chemo target agents significantly improved the disease control rate.	[29]
20 advanced cancer patients with metastases.	A prospective, open-label clinical study.	Glycosidase-digested fucoidan extracted from *C. okamuranus.*	Anti-inflammatory effects of fucoidans. Patients’ quality of life stayed almost stable without significant changes.	[30]
20 patients with unresectable advanced or recurrent colorectal cancer scheduled to undergo treatment with FOLFOX or FOLFIRI protocols.	A randomized trial.	*C. okamuranus*.	Chemotherapy with fucoidan was continued for a longer period than chemotherapy without fucoidan.	[31]
10 patients with cancer of stage IIa-IV.	Case reports.	Fucoidan, derived from *C. okamuranus.*	Increase in tumor immunity and lessening of the pain symptoms.	[32]
100 patients with locally advanced rectal cancer.	A double-blind, randomized, placebo-controlled, parallel study.	Fucoidan (without clarification).	No results posted.	[141]
119 patients with squamous cell carcinoma.	A randomized, double-blind study.	Dietary fucoidan supplement.	Study not completed.	[142]
100 patients with advanced hepatocellular carcinoma.	A randomized, double-blind study controlled trial.	Dietary fucoidan supplement.	Study not completed.	[143]
Patients with non-small cell lung cancer.	A double-blind randomized controlled trial.	Dietary supplement oligo fucoidan	Withdrawn.	[144]
39 volunteers.	A randomized, double-blind, parallel-group, placebo-controlled pilot study	Okinawa mozuku-derived fucoidan	NK cell activity was significantly enhanced.	[145]
45 patients with chronic gastritis.	A double-blind placebo-controlled study.	Combination of wheat peptides and fucoidan.	Mitigated the progression of chronic gastritis, altering gut microbial profile, and short chain fatty acids production.	[78]

**Figure 2:**
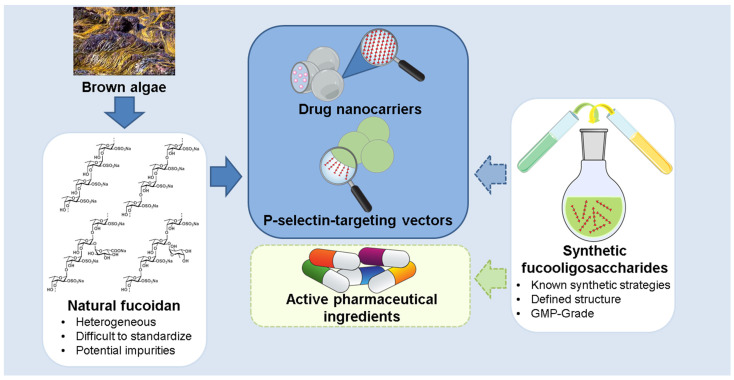
Perspectives and challenges in fucoidan application.

So far, the accumulated data suggest that our knowledge of fucoidan structures and activities is limited because of their very broad structural variability. Indeed, they may contain components with non-fucose residues in side chains [146] and the backbone [147], and can also include not only fucopyranose units but even fucofuranose ones [148]. The sets of these polysaccharides can be regarded as libraries to screen potential active hits for their further structural standardization. It has been suggested that the anticancer effect of fucoidans is connected with their ability to inhibit the interaction of P-selectin, growth factors, and other tumor growth-associated receptors with their cellular ligands [46,58,79,80]. Particularly, fucoidans may mimic sialylated oligosaccharides which are critically important fragments of ligands recognized by P-selectin. Since the reparation of sialylated oligosaccharide by chemical (see for example [149,150,151]) or by biotechnology methods [152] is a non-trivial task, fucoidans can be used as a starting point for the rational design of new drugs related to fucoidans but having well-characterized and standardized structures. They can potentially be prepared by using fucoidan-degrading and modifying enzymes [153,154,155], but as of now, only a few agents of this type have been obtained in practical amounts, while their industrial applicability is yet to be investigated. Chemical synthesis of fucoidan-related oligosaccharides of desirable structure, meanwhile, is practically possible today using already well-elaborated methods [156,157,158,159]. Thus we can actually expect the appearance of fucoidan-related drugs as agents for supportive care and symptomatic therapy in cancer patients in the future (Figure 2). The use of such drugs after chemo- or radiation therapy might contribute to the recovery of hematopoiesis, while their anti-inflammatory, immunomodulatory, and anticoagulant functions stand to improve the quality of life of patients with advanced cancers.

## Data Availability

Not applicable.
